# Association of parental prenatal mental health with offspring neurodevelopmental disorders: a systematic review and meta-analysis

**DOI:** 10.1017/S0033291725103139

**Published:** 2026-01-20

**Authors:** Adrianna Kępińska, Thalia Robakis, Shelby Smout, Rachel Bercovitch, Lily Cohen, Ingrid Christina Gustavsson Mahjani, Alkistis Skalkidou, Veerle Bergink, Behrang Mahjani

**Affiliations:** 1 Icahn School of Medicine at Mount Sinai, USA; 2 Uppsala Universitet, Sweden; 3 Karolinska Institutet, Sweden

**Keywords:** anxiety disorders, attention-deficit/hyperactivity disorder, autism, intellectual disability, maternal, mood disorder, neurodevelopmental disorders, obsessive–compulsive disorder, paternal, prenatal, maternal effect, paternal effect

## Abstract

**Background:**

Parental prenatal mood and anxiety disorders (PMADs) are linked to child neurodevelopmental disorders (NDDs), but evaluations of the magnitude and mechanisms of this association are limited. This study estimates the strength of the association and whether it is impacted by genetic and environmental factors.

**Methods:**

A systematic search of PubMed, CENTRAL, PsycINFO, OVID, and Google Scholar was performed for articles published from January 1988 to September 2025. Of 2,420 articles screened, 74 met the inclusion criteria. Meta-analyses were conducted on 21 studies, and 53 were included in the narrative synthesis. We conducted random-effects meta-analyses, along with tests for heterogeneity (*I* ^2^) and publication bias (Egger’s test). The review followed PRISMA and MOOSE guidelines.

**Results:**

Maternal PMADs were associated with a significantly increased risk of attention-deficit/hyperactivity disorder (ADHD; odds ratio [OR] 1.91, 95% confidence interval [CI] 1.45–2.52) and autism spectrum disorder (ASD; OR 1.75, 95% CI 1.43–2.14) in children. Paternal PMADs were also associated with the risk of NDDs, with combined odds for ASD and ADHD (OR = 1.23, 95% CI 1.14–1.33). Several studies suggested that the link between parental PMADs and offspring NDDs might be impacted by both genetic and environmental factors, including the impact of ongoing parental depression on child behavior.

**Conclusions:**

Parental PMADs are associated with increased risk of NDDs in children. These findings likely reflect a combination of inherited liability and environmental processes; clarifying mechanisms will require genetically informed designs. Regardless of mechanism, offering optional, family-centered developmental support may help promote child well-being in families where a parent is experiencing PMADs.

## Introduction

As of 2021, 8.56% of children in the United States have a neurodevelopmental disorder (NDD), including autism spectrum disorder (ASD), intellectual disability (ID), and attention-deficit/hyperactivity disorder (ADHD; Boyle et al., 2011; Zablotsky et al., 2023). Recognizing factors that predispose individuals to NDDs and enhancing early detection can lead to interventions that reduce the severity of NDD symptoms and improve the quality of life for neurodivergent individuals and their families (Aldharman et al., 2023). Furthermore, timely interventions can help prevent the progression of these conditions into other severe mental or medical complications in adulthood.

Recent research has demonstrated a significantly increased risk of NDDs in children born to mothers who experienced mood or anxiety disorders during pregnancy (El Marroun et al., 2014; Kodesh et al., 2021). This finding highlights the critical implications of maternal mental health on early neurodevelopmental outcomes and underscores the importance of integrating maternal health considerations into early pediatric care to support healthy developmental trajectories. Additionally, this body of research suggests a shared familial liability between ASD, ADHD, anxiety, and mood disorders, indicating that genes inherited from parents may predispose children to NDDs. It is also possible that genetic predispositions could interact with environmental factors such as maternal mental health to influence the development and severity of NDDs in children, thus necessitating a multifaceted approach in research and intervention strategies that consider both genetic and environmental contributions.

Despite extensive investigation into maternal mental health, such as prenatal depression, significant gaps remain in our understanding, particularly the magnitude of the association and specific mechanisms through which prenatal mood and anxiety disorders (PMADs) affect neurodevelopmental outcomes. Additionally, although maternal influences have been extensively studied, the potential effects of paternal mental health remain largely unexplored. This oversight represents a critical gap in our knowledge, given the potential influence of paternal mental health and genetic contribution on developmental trajectories.

To address these gaps, we conducted a systematic review and meta-analysis of studies exploring the associations between maternal and paternal PMADs, specifically prenatal depression, anxiety disorders, and obsessive–compulsive disorder (OCD), and the risk of NDDs in offspring. This emphasis on the prenatal period is crucial, given its relatively limited coverage in existing literature compared with the broader perinatal and early postnatal periods. For NDDs, our focus was on three major conditions: ASD, ADHD, and ID, due to their shared genetic and phenotypic overlaps. For studies not suitable for meta-analysis, we performed narrative syntheses to provide a comprehensive overview of existing literature. For the purposes of this study, we define ‘maternal’ as relating to the parent who carried the pregnancy and ‘paternal’ as relating to the other biological parent.

A crucial aspect of our study is analyzing whether the association between PMADs and NDDs in offspring primarily stems from shared genetic variation. We consider the possibility that this association may not be directly due to the environmental or behavioral influences of having a parent with PMADs but could predominantly result from genetic predispositions passed from parents to children. Understanding this potential genetic basis is critical for discerning whether the observed association reflects modifiable risk factors or primarily represents inherited genetic vulnerability.

## Methods

### Search Strategy

**Figure 1.**
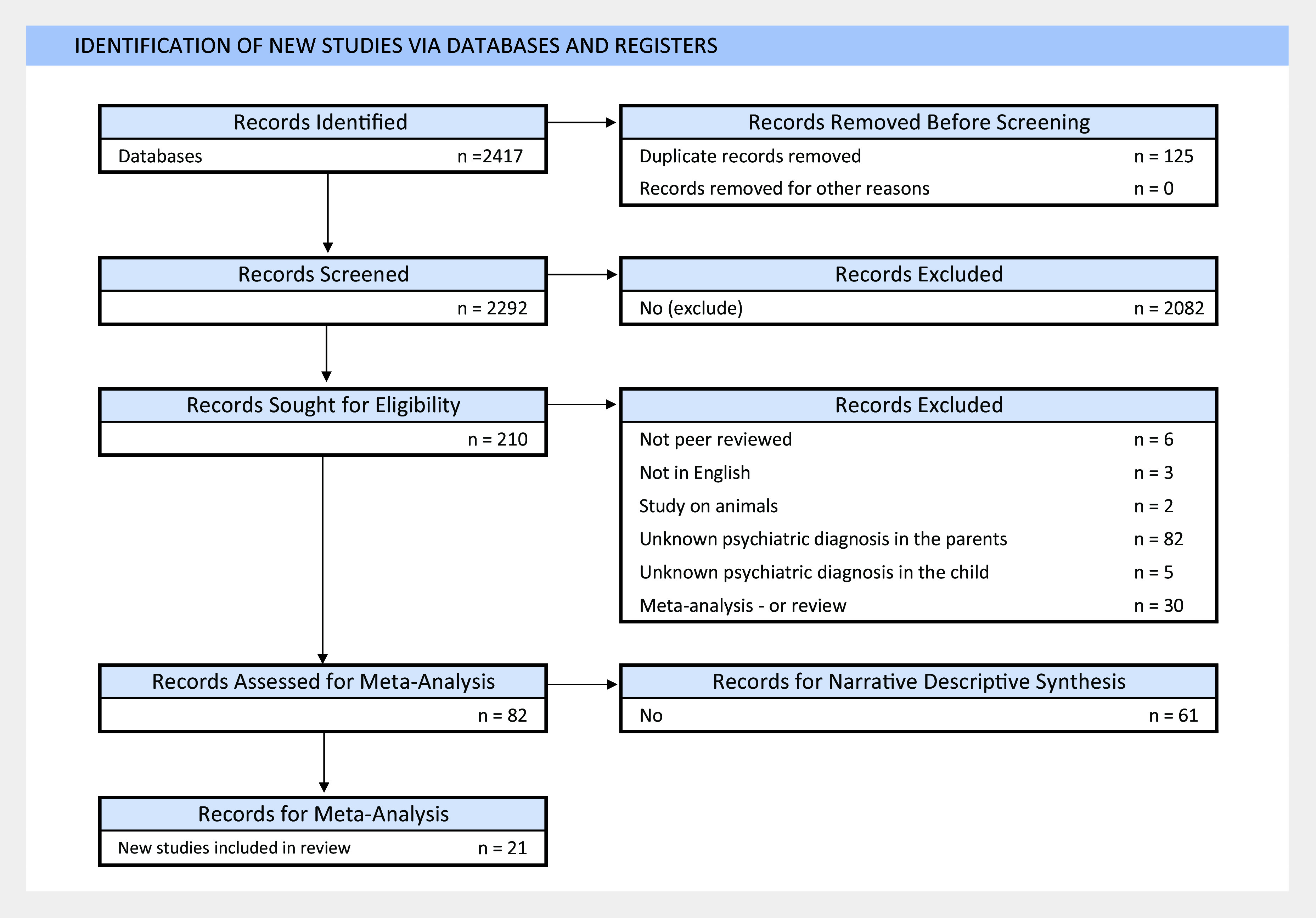
PRISMA flow diagram.

This systematic review and meta-analysis followed recommendations of the Preferred Reporting Items for Systematic Reviews and Meta-Analyses (PRISMA; Supplementary Table S1) and of Meta-Analysis of Observational Studies in Epidemiology (MOOSE) guidelines (Moher et al., 2010). The study protocol is registered with PROSPERO (ID: CRD42022370757). Figure 1 outlines our article selection process, including identification, screening, and eligibility assessment. Two researchers completed searches (L.E.C. and B.M.).

#### Inclusion criteria

Studies on maternal/paternal PMADs and offspring NDDs were sought from PubMed, Cochrane CENTRAL, OVID, and Google Scholar, covering January 1, 1988, to September 20, 2025 (see Supplementary Table S2 for search terms and details). We selected this study period to ensure comprehensive coverage of recent literature. We included cohort, case–control, and cross-sectional studies.

We defined PMADs as depressive disorders, anxiety disorders, and OCD. While OCD is classified separately from anxiety disorders in the *Diagnostic and Statistical Manual of Mental Disorders, Fifth Edition* (DSM-5), we included it a priori given its frequent co-study with anxiety in perinatal research. Where feasible, we report OCD findings separately.

#### Exclusion criteria

We excluded case studies, ecological/animal models, qualitative studies, and psychometric studies of PMADs and NDDs due to organic brain syndrome, substance use, or known physiological conditions. Because the goal of this study is to assess the relationship between parental PMADs and offspring NDDs, these studies were excluded to limit the number of confounding variables and pathophysiological mechanisms that could moderate this relationship. We also excluded gray literature, unpublished, not English-language research, and studies with fewer than 20 participants.

#### Screening

L.E.C. and B.M. independently screened titles and abstracts to remove duplicates and irrelevant studies using DistillerSR software (Evidence Partners, Ottawa, ON, Canada).

#### Eligibility

Full texts from screening and references from review articles were assessed independently by A.P.K. and L.E.C., with discrepancies resolved by discussion and random rescreening by B.M. Articles were categorized based on whether they had relevant data for either the meta-analysis or narrative descriptive synthesis. The data required for inclusion in the meta-analysis are described in the following text.

### Data extraction

Data extractions were independently conducted by three researchers (A.P.K., L.E.C., and B.M.), with disagreements resolved through full-text review. Extracted data included the following: participant numbers with and without parents with PMADs and with and without NDDs; where available, reported measures of effect (odds ratios unadjusted for covariates [ORs], 95% confidence intervals [CIs]) and directions of effect; publication year; parental exposure to antidepressants; timing of parent exposure; parent age; parent exposure measures; offspring outcome (NDD diagnosis or symptoms); offspring age; sample size; number of offspring per sex/gender; setting; study/cohort/register name (where applicable); and participant country of origin, ethnicity, race, or ancestry (as reported).

Exposure timing was coded as preconception, prenatal, or postnatal, as reported in each study. Preconception assessments (e.g. diagnoses recorded in the year before pregnancy) were explicitly noted in Table 1 for maternal studies and Table 2 for paternal studies, as well as in Supplementary Table S1.

**Table 1.** Characteristics of meta-analyzed studies on maternal prenatal mood and anxiety disorders and offspring neurodevelopmental disorders

Study	Maternal exposure	Mother exposure assessment time	Exposure measures	Offspring outcome	Outcome measures	Study design	Sample size	Setting
Grzeskowiak et al. (2016)	Antidepressants; antenatal mood	6–10, 17–32 weeks of gestation	SCL-8d, self-reported use of drugs from ATC code class N06A	Behavioral problems: emotional; conduct; peer relationship; hyperactivity/inattention; pro-social skills	SDQ >90th percentile	Nationwide population-based	48,737	Denmark
Tusa et al. (2025)	Depression	Conception to delivery	ICD-10	ASD	ICD-10	Retrospective cohort	223,068	Australia
Lupattelli et al. (2022)	SSRI and SNRI antidepressants; depression or anxiety	Weeks 17 and 30 of pregnancy; offspring age 6 months to adolescence	Self-reported symptoms; SCL-5; medication use self-report; NorPD filled antidepressant prescriptions	ADHD	ICD-10; dispensed ADHD medications; CPRS-R, *z*-scores, higher scores indicate greater ADHD symptoms	Nationwide, prospective population-based pregnancy	6,395	Norway
Bolea-Alamañac et al. (2019)	Somatic anxiety symptoms	Weeks 18 and 32 of pregnancy	CCEI scores: top 20% for case status	Hyperactivity/inattention symptoms; ADHD diagnosis	SDQ scores >2 SD above mean; DAWBA bands 4–5 high ADHD probability	Population-based cohort	11,029	United Kingdom
D’Souza et al. (2019)	Depression; anxiety	Antenatal (exact timing NR) and 9 months postnatal	EPDS ≥13; PSS; GAD-7 scores, moderate to severe anxiety (10–21)	Emotional symptoms; peer problems; hyperactivity-inattention; conduct problems; total difficulties	SDQ Preschool ranges: top 10% ‘abnormal’	Longitudinal prospective	6,246	New Zealand
Nidey et al. (2021)	Depression	1 year prior to pregnancy up to delivery	ICD-9/10	ADHD	ICD-9/10	Population-based retrospective cohort	5,635	United States
Huhdanpää et al. (2021)	Depression	32nd pregnancy week and at offspring age 3, 8, and 24 months	CES-D	Inattentive and hyperactive symptoms of ADHD	SDQ: inattentive/hyperactive symptoms ≥5 (75th percentile); FTF: inattention and hyperactivity-impulsivity domains ≥6 (75th percentile)	Population-based birth	699	Finland
Hagberg et al. (2018)	Depression; antidepressant prescription	Within 1 year before delivery	Read codes from CPRD (diagnostic criteria NR)	ASD; Asperger’s syndrome; PDD	Read diagnostic codes	Cohort	194,494	United Kingdom
Clements et al. (2015)	Antidepressants at preconception and during pregnancy; depression	Pregnancy trimesters or at any time before pregnancy	Outpatient EHR prescriptions; inpatient pharmacy dispenses; ICD-9	ASD; ADHD	ICD-9; DSM-IV	Cohort	13,273	United States
Chien et al. (2023)	Depressive, anxiety, and obsessive–compulsive disorders	Before childbirth; between birth and offspring ASD diagnosis; after offspring ASD diagnosis	ICD-9/ICD-10-CM	ASD	ICD-9-CM/10	Population-based case–control study	121,994	Taiwan
Kodesh et al. (2021)	Depression	636 days prior to the offspring’s date of birth	ICD-9	ASD	ICD–9/10	Exploratory case-cohort, population-based	80,187	Israel
Rai et al. (2013)	Depression; SSRIs and NSMRIs during pregnancy	Depression onset/length NR; SSRI/NSMRI use at pregnancy median 10 weeks	ICD-10 diagnoses only before childbirth; medications with ATC code N06AB and N06AA	ASD (with and without ID)	ICD-9/10; DSM-IV	Population-nested case–control	43,277	Sweden
El Marroun et al. (2014)	Depressive symptoms; prenatal SSRI use	Average 20.6 weeks of pregnancy	BSI >0.75; CIDI; self-reported medication use; pharmacy prescription records	Autistic symptoms: interpersonal behavior; communication; repetitive/stereotypic behaviors	CBCL 1.5–5: clinically meaningful problems >93rd percentile; pervasive developmental problems >95th percentile; affective problems >89th percentile; SRS	Population-based cohort	5,976	The Netherlands
Avalos et al. (2023)	Depression	4 weeks preconception to 8 weeks postpartum	Diagnosis self-report; ICD-9/10; PROMIS-D T-scores (continuous and ≥ 65.9 severe depression)	ASD-related traits	SRS *T*-scores (continuous and SRS *T*-score ≥66 moderate-to-severe autism-related traits)	Longitudinal collaborative cohort	5,553	United States
Leis et al. (2014)	Depression and anxiety	18 and 32 weeks of pregnancy; 8 weeks, 8, 21, 33, 61, 73 months, and 11 years postpartum	EPDS ≥13; CCEI ≥10	Conduct problems, emotional symptoms, hyperactivity, peer relationships, and prosocial behavior	SDQ, summed to subscale scores, range 0–10	Prospective, community-based	2,891	United Kingdom
Nishigori et al. (2023)	Psychological distress; depressive moods and anxiety	Median 15.1 (IQR 12.3–18.9); median 27.4 (IQR 25.3–30.1) weeks of pregnancy	K6 ≥ 5	ASD	ICD-10	Nationwide prospective birth cohort	78,745	Japan
Seebeck et al. (2024)	Depression; antidepressants	Mean 35.2 (SD 1.5) weeks of pregnancy	EPDS, higher scores indicate higher depression levels; self-reported antidepressant use	ASD risk	SSI-T <45	Prospective cohort	2,388	United States
Siracusano et al. (2021)	Depression; psychotropic medications during pregnancy	First or second pregnancy trimester	DSM-5; EPDS ≥12; psychotropic medication prescription	ASD; hyperactivity and inattention	DQ of Griffiths III; WISC-IV, standardized; ABAS-II; DSM-5; ADOS-2; CBCL, *T* ≥ 70 for clinically significant; Conners’ Parents Rating Scale–Long Form, *T* ≥ 70 for clinically significant	Cohort	59	Italy
Hope et al. (2024)	Depression	Between minimum 2 years, 280 days and 280 days before birth	CPRD read codes	ADHD/ADD; ASD; ID; cerebral palsy; epilepsy	CPRD prescriptions; HES ICD CPRD read codes; ICD-10	Population	410,461	England

*Note*: Participants are described as mothers to reflect the language used in meta-analyzed papers; a single study (Avalos et al., 2023) defined how parents were ascertained and identified them as birthing parents. ATC, Anatomical Therapeutic Chemical Classification System; ADHD, attention-deficit hyperactivity disorder; ICD-10, International Classification of Diseases, Tenth Revision; SCL-8d, eight-item Symptom Checklist version; SDQ, Strengths and Difficulties Questionnaire; EHR, electronic health records; ASD, autism spectrum disorder; ICD-9, International Classification of Diseases, Ninth Revision; DSM-IV, *Diagnostic and Statistical Manual of Mental Disorders, Fourth Edition*; SSRI, selective serotonin reuptake inhibitors; SNRI, serotonin-norepinephrine reuptake inhibitors; SCL-5, Symptom Checklist-5 five-item version; NorPD, Norwegian Prescription Database; CPRS-R, Conners’ Parent Rating Scale–Revised; CCEI, Crown-Crisp Experiential Index; DAWBA, Development and Well-Being Assessment; EPDS, Edinburgh Postnatal Depression Scale; PSS, Perceived Stress Scale; GAD-7, General Anxiety Disorder–7; CPRD, Clinical Practice Research Datalink; PDD, pervasive developmental disorder; ICD-9-CM, International Classification of Diseases, Ninth Revision, Clinical Modification; ICD-10-CM, International Classification of Diseases, Tenth Revision, Clinical Modification; PROMIS-D, Patient-Reported Outcomes Measurement Information System–Depression. SRS, Social Responsiveness Scale; BSI, Brief Symptom Inventory; CIDI, Composite International Diagnostic Interview; CBCL, Child Behavior Checklist; K6, Kessler Psychological Distress Scale; SSI-T, Social Security Income–Test; DQ, Developmental Quotient; WISC-IV, Wechsler Intelligence Scale for Children–Fourth Edition; ABAS-II, Adaptive Behavior Assessment System–Second Edition; ADOS-2, Autism Diagnostic Observation Schedule–Second Edition; HES, Hospital Episode Statistics.

a Studies by Bolea-Alamañac et al. (2019) and Leis et al. (2014) use the same sample but address different offspring outcomes. Consequently, data from these studies were preserved for meta-analysis.

**Table 2.** Characteristics of meta-analyzed studies on paternal prenatal mood and anxiety disorders and offspring neurodevelopmental disorders

Study	Paternal exposure	Parent exposure assessment time	Exposure measures	Offspring outcome	Outcome measures	Study design	Sample size	Setting
Van Batenburg-Eddes et al. (2013)	Anxiety and depression	Generation R: 20 weeks of pregnancy; ALSPAC: 18 weeks of pregnancy; 33 months postnatally	Generation R: BSI; ALSPAC: EPDS; CCEI	Attention problems	Generation R: the Child Behavior Checklist, 93rd percentile for Attention Problems in the borderline range; ALSPAC: SDQ: hyperactivity/inattention subscale, in the ‘abnormal’ range	Cross-cohort	Generation R: 2,280; ALSPAC: 3,442	United Kingdom; The Netherlands
Ramchandani et al. (2008)	Depression	Week 18 of their partners’ pregnancy; 8 weeks after birth	EPDS ≥13	Emotional problems; conduct problems; hyperactivity; prosocial behavior; DSM-IV psychiatric diagnoses	Rutter Revised Preschool Scales, top 10% for high-scorers; DAWBA for DSM-IV psychiatric diagnoses	Longitudinal population cohort	7,601	United Kingdom
Huhdanpää et al. (2021)	Depression	32nd pregnancy week and at offspring age 3, 8, and 24 months	CES-D	Inattentive and hyperactive symptoms of ADHD	SDQ: inattentive/hyperactive symptoms ≥5 (75th percentile); FTF: inattention and hyperactivity-impulsivity domains ≥6 (75th percentile)	Population-based birth	699	Finland
Chien et al. (2023)	Depressive, anxiety, and obsessive–compulsive disorders	Before childbirth; between birth and offspring ASD diagnosis; after offspring ASD diagnosis	ICD-9/ICD-10-CM	ASD	ICD-9-CM/10	Population-based case–control study	121,994	Taiwan
Rai et al. (2013)	Depression; SSRIs and NSMRIs during pregnancy	Depression onset/length NR; SSRI/NSMRI use at pregnancy median 10 weeks	ICD-10 diagnoses only before childbirth; medications with ATC codes N06AB and N06AA	ASD (with and without ID)	ICD-9/10; DSM-IV	Population-nested case–control	43,277	Sweden

*Note*: Participants are described as fathers to reflect the language originally used in meta-analyzed papers. ATC, Anatomical Therapeutic Chemical Classification System; ADHD, attention-deficit/hyperactivity disorder; ICD-10, International Classification of Diseases, Tenth Revision; ALSPAC, Avon Longitudinal Study of Parents and Children; ICD-9, International Classification of Diseases, Ninth Revision. ICD-9-CM, International Classification of Diseases, Ninth Revision, Clinical Modification; ICD-10-CM, International Classification of Diseases, Tenth Revision, Clinical Modification; DSM-IV, *Diagnostic and Statistical Manual of Mental Disorders, Fourth Edition*; SSRI, selective serotonin reuptake inhibitor; SNRI, serotonin-norepinephrine reuptake inhibitor; SDQ, Strengths and Difficulties Questionnaire; ASD, autism spectrum disorder; EPDS, Edinburgh Postnatal Depression Scale; DAWBA, Development and Well-Being Assessment; CCEI, Crown-Crisp Experiential Index; CES-D, Center for Epidemiologic Studies Depression Scale.

a Van Batenburg-Eddes et al. (2013) analyzed data from both mothers and fathers but only the data from the fathers were included in the meta-analysis as the study sample for mothers overlaps with a larger study on maternal perinatal disorder by Bolea-Alamañac et al. (2019).

Although our study primarily addressed the association of PMADs and NDDs, several studies examined the influence of prenatal antidepressant exposure. We only meta-analyzed or reviewed findings where antidepressants were ascertained as a treatment for PMADs and not other conditions, such as migraines and sleep disorders (Supplementary Table S1).

Suggested text for the Methods section: Our meta-analysis included studies using both clinical diagnoses (ICD/DSM criteria from medical records or clinical interviews) and validated symptom measures from community samples. For parental PMADs, studies varied between diagnosed mood and anxiety disorders and symptom scales above established clinical thresholds (e.g. Edinburgh Postnatal Depression Scale [EPDS] ≥13, State–Trait Anxiety Inventory scores in the clinical range). For offspring NDDs, we included both formal clinical diagnoses and elevated scores on validated diagnostic screening instruments (e.g. Strengths and Difficulties Questionnaire [SDQ] scores above clinical cutoffs). While we prioritized clinical thresholds where available for symptom-based measures, we acknowledge that heterogeneity in measurement approaches (clinical diagnoses vs. symptom scales) may contribute to the observed statistical heterogeneity in our pooled estimates.

### Statistical analysis

Our meta-analysis included parents diagnosed with mood or anxiety disorders during pregnancy or both prenatally and postnatally, excluding those diagnosed only postnatally. Where multiple studies used the same cohort, we selected the largest one to avoid sample overlap. We conducted random-effects meta-analyses using R packages *meta* and *dmetar* to account for error variance within and between studies (Companion R package for the guide Doing Meta-Analysis in R, 2024; Viechtbauer, 2010). Effect sizes (OR) and variances were required for each study. For studies with counts of exposed and unexposed participants, parameters were calculated using the *meta metabin* function. When only unadjusted ORs and confidence intervals were available, variance was derived using the standard formula to convert CI width to standard error.

We assessed study heterogeneity with Cochran’s *Q* test and the *I* ^2^ statistic and identified outliers using funnel plots. Publication bias was evaluated using the trim-and-fill method (Shi & Lin, 2019). Maternal and paternal findings were analyzed separately. We stratified analyses by offspring NDD for maternal studies only, as the number of paternal studies was insufficient for stratification. Sensitivity analyses were conducted by excluding studies identified as potential outliers through funnel plot inspection.

Studies with information insufficient for meta-analysis were included in a narrative synthesis (see Supplementary Table S3 for methods). Reasons for insufficiency included when effect sizes could not be harmonized for meta-analysis, for example, when only regression coefficients or correlations were reported, when outcomes were symptoms rather than diagnoses, when parental PMADs were not distinguished by mother versus father, or when the design focused on genetic/mechanistic measures such as polygenic risk scores (PRSs).

Two researchers (A.P.K. and B.M.) independently assessed the risk of bias in all studies using the Newcastle–Ottawa scales and the Joanna Briggs Institute (JBI) Critical Appraisal Checklists (Supplementary Table S4; Aromataris & Munn, 2020; Barker et al., 2023; Wells et al., 2011). The authors resolved any disagreements through discussion.

## Results

A total of 2,420 studies initially met the inclusion criteria. We selected 74 studies for the systematic review and 21 for meta-analysis (Figure 1).

Characteristics of meta-analyzed studies are detailed in Tables 1 and 2. Across all included studies, 35 reported participant race, country of origin, and ancestry or ethnicity, but none specified how these demographics were ascertained. The studies varied in their designs and implemented measurement tools (Tables 1 and 2; additional details in Supplementary Table S1).

Beyond the 21 studies included in meta-analysis, we conducted a comprehensive narrative synthesis of the remaining 53 studies that were unsuitable for meta-analysis due to insufficient data for pooling.

### Maternal PMAD and the risk of offspring NDDs

**Figure 2.**
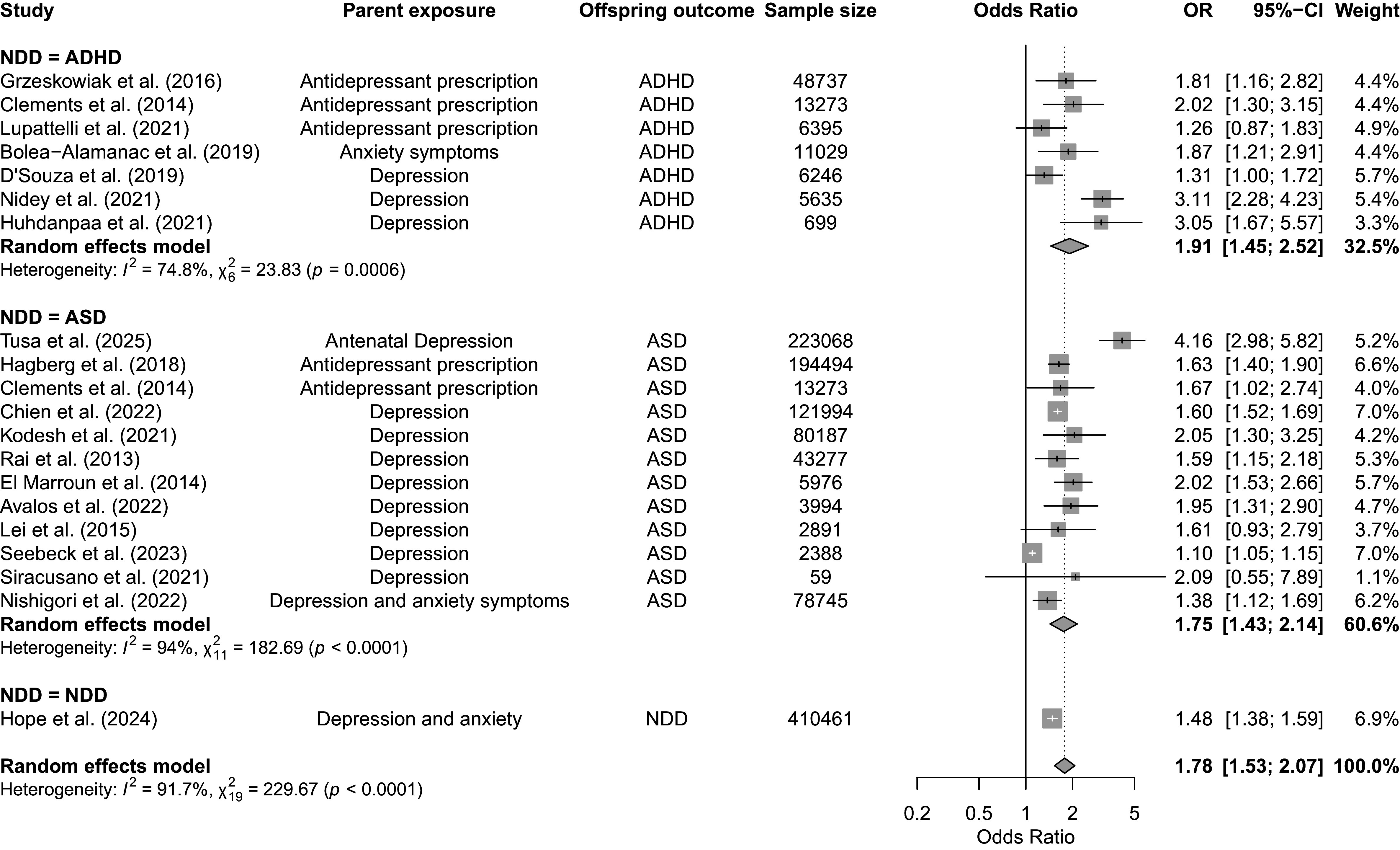
Forest plot of associations between maternal prenatal mood and anxiety disorders and offspring neurodevelopmental disorders. *Note*: Square sizes reflect the weights attributed to each study. Diamonds denote the summary effect sizes for the random-effects models. OR, odds ratio; CI, confidence interval; NDD, neurodevelopmental disorder; ADHD, attention-deficit/hyperactivity disorder; ASD, autism spectrum disorder. Clements et al. (2015) analyzed separate samples of individuals with diagnoses of autism spectrum disorder and attention-deficit/hyperactivity disorder. The authors provided results for three trimesters. The third trimester has been selected for this meta-analysis because it is the most conservative estimate reported. Chien et al. (2023) included the following disorders: major depressive disorder; persistent depressive disorder; and depressive disorder, unspecified. Hope et al. (2024) analyzed a combined sample of individuals with any of the following disorders: autism/autism spectrum disorder, attention-deficit/hyperactivity disorder, intellectual disability, cerebral palsy, and epilepsy. For Bolea-Alamañac et al. (2019), maternal anxiety was measured at 18 and 32 weeks’ gestation. The final variable analyzed was a composite of both assessments, with somatic anxiety dichotomized at the 80th percentile.

Our meta-analysis of 20 articles revealed a significant association between maternal PMADs and offspring NDDs (OR = 1.78, 95% CI 1.53–2.07, *p* < 0.0001; Figure 2). Visual inspection of the funnel plot (Supplementary Figure S1) suggested asymmetry, with several studies appearing as potential outliers (Nidey et al., 2021; Seebeck et al., 2024). Formal outlier screening using dmetar::find.outliers (DerSimonian-Laird *τ* ^2^; Hartung-Knapp CIs) identified three studies for exclusion in sensitivity analyses (Nidey et al., 2021; Seebeck et al., 2024; Tusa et al., 2025). Removing these studies reduced heterogeneity from high to low (Cochran’s *Q* = 229.67, *p* < 0.0001 to *Q* = 20.63, *p* = 0.1930, *I* ^2^ = 91.7% to *I* ^2^ = 22.5%) and the OR to 1.59 (95% CI 1.50–1.68; Supplementary Figure S2; Higgins, 2003). These sensitivity results indicate that the pooled association is not driven by a small number of outlying studies.

Egger’s test applied to the full dataset suggested funnel plot asymmetry (*t* = 2.63, *p* = 0.017), but this result coincided with extreme between-study heterogeneity (*τ* ^2^ = 9.22). After excluding the three prespecified outliers, the Egger test was no longer significant (*t* = 1.57, *p* = 0.14), and heterogeneity was substantially reduced (*τ* ^2^ = 1.18). These findings indicate that the apparent asymmetry in the unfiltered analysis was likely driven by outliers, rather than systematic publication bias. In support of this result, the trim-and-fill method estimated only one potentially missing study (Supplementary Figure S3).

#### Effects of maternal PMADs on offspring ADHD

Our meta-analysis of seven articles revealed a significant association between maternal PMADs and an increased risk of offspring ADHD (OR = 1.91, 95% CI 1.45–2.52; Figure 2). Initially, the ADHD model exhibited high heterogeneity (Cochran’s *Q* = 23.83, *p* = 0.0006, *I* ^2^ = 74.8%). After the exclusion of the three outlier studies, heterogeneity decreased (Cochran’s *Q* = 9.90, *p* = 0.08, *I* ^2^ = 49.5%), and OR changed to 1.70 (95% CI 1.34–2.14; Supplementary Figure S1; Higgins, 2003).

Narrative evidence is consistent with these findings. Several studies reported associations between maternal PMADs and ADHD diagnoses or symptoms in offspring (Shuffrey et al., 2023). Mechanistic work suggests inflammatory pathways may be involved: in one prospective cohort, first-trimester elevations in CRP, IL-1β, and MCP-1 partially mediated (~12%) the association between maternal depressive symptoms and poorer infant adaptive functioning, whereas higher IL-10 predicted better personal-social outcomes.

Findings on why this association occurs are mixed. One study using PRSs for ADHD found that part of the association with prenatal depression was attributable to shared genetic (Chen et al., 2020) liability. Other work, however, found persistent associations even after adjusting for genetic risk (Eilertsen et al., 2021). Epigenetic analyses add further nuance: cord blood DNA methylation was not associated with maternal prenatal depression or offspring ADHD symptoms (Bendiksen et al., 2020; Betts et al., 2014, 2015; Huhdanpää et al., 2021; Kingston et al., 2018; Koutra et al., 2017; Lahti et al., 2017; MacKinnon et al., 2018; O’Donnell et al., 2017; Olstad et al., 2023; Park, 2018; Teyhan et al., 2014; Van Batenburg-Eddes et al., 2013; Wolford et al., 2017). Maternal anxiety has also been linked with offspring ADHD, although results are inconsistent regarding sensitive exposure periods. Some studies suggest early pregnancy anxiety predicts risk, whereas others emphasize gene–environment interactions later in gestation. More recent genetically informed studies support the contribution of both inherited and environmental factors. In one longitudinal cohort with independent replication, prenatal depression predicted ADHD and emotional symptoms independently of child PRSs, whereas child PRS effects grew stronger with age. Complementary trio-based analyses found strong effects of transmitted PRSs but limited evidence for non-transmitted (‘genetic nurture’) influences. Together, these studies suggest inherited liability is important, but maternal environmental exposures exert independent influence.

Two cross-cutting considerations emerged across cohorts. First, effect sizes vary by informant and measurement, with larger associations often reported for parent-rated ADHD symptoms and smaller or more variable effects for teacher reports or clinical diagnoses, consistent with known informant and ascertainment differences. Second, associations are consistently observed for preschool and early-childhood symptom measures, whereas findings at later ages and for diagnostic outcomes are more heterogeneous across cohorts and instruments (e.g. Kingston et al., 2018; Lahti et al., 2017; Lin et al., 2024; Park, 2018; Wolford et al., 2017).

Trajectory studies provide additional insights. Persistently high or increasing maternal depression during pregnancy is associated with elevated ADHD symptoms in offspring, whereas consistently low or decreasing symptoms predict fewer difficulties. This pattern suggests that both timing and severity of maternal depression shape ADHD trajectories. Recent register studies further highlight timing, with prenatal depression linked to broader outcomes than depression first recorded postnatally.

Accounting for postnatal maternal symptoms, several cohorts explicitly tested whether postnatal maternal psychopathology explained the prenatal association. In PREDO, prenatal depressive symptoms remained associated with child ADHD at 3–6 years after adjustment for postnatal maternal depression, although the effect attenuated (Wolford et al., 2017). In ALSPAC and Generation R, prenatal maternal depression/anxiety predicted attention/hyperactivity, but these associations were substantially reduced – and in Generation R became nonsignificant – after adding maternal symptoms measured ≈33 months postpartum (Van Batenburg-Eddes et al., 2013). Together, these findings indicate that postnatal symptomatology contributes to the observed association but does not fully account for it, consistent with additive prenatal and postnatal influences.

Comparisons of depression and anxiety exposures suggest possible differences: maternal anxiety may be more strongly associated with hyperactivity, whereas maternal depression more consistently predicts a broader range of ADHD symptoms. However, inconsistent findings across studies likely reflect differences in study design, measurement, and sample size.

Finally, new work emphasizes the importance of comorbidity. In one prospective perinatal cohort, maternal ADHD symptoms amplified the effects of maternal depression: depressive symptoms predicted toddler emotional and attentional difficulties only at higher levels of ADHD. This suggests that comorbidity identifies subgroups of children at particularly high risk, a nuance not addressed in earlier studies.

#### Effects of maternal PMADs on offspring ASD and ID

Our meta-analysis of 12 studies revealed an association between maternal PMADs and an increased risk of offspring ASD (OR = 1.75, 95% CI 1.43–2.15). Studies initially presented with high heterogeneity (Cochran’s *Q* = 182.69, *p* < 0.0001, *I* ^2^ = 94%), but upon removal of the three aforementioned outliers in the overall analysis, heterogeneity was low (Cochran’s *Q* = 6.96, *p* = 0.64, *I* ^2^ = 0%). The pooled OR decreased to 1.61 (95% CI 1.54–1.69; Supplementary Figure S2; Seebeck et al., 2024), indicating that the heterogeneity, rather than the association, was driven by a few extreme estimates.

Large-scale register studies published since 2024 further corroborate these associations. In a Taiwanese national cohort, parental major depression was associated with increased hazards of offspring (Connor et al., 2022) ASD (HR 1.52, 95% CI 1.16–1.94), alongside ADHD, tic disorder, and developmental delay, with broader associations for prenatal compared with postnatal exposure. In an Australian retrospective cohort study, both antenatal and postnatal maternal depressive disorders were linked to higher ASD risk (adjusted relative risks [RRs] 1.61 and 1.85, respectively), with mediation by preterm birth and low Apgar scores accounting for only ~1% of the association (Tusa et al., 2025). These findings suggest largely direct pathways from maternal mood disorders to offspring ASD.

Accounting for postnatal maternal symptoms, few studies explicitly adjusted for or separated prenatal from postnatal maternal psychopathology in the context of ASD; among those that did, both periods were associated with risk, and prenatal associations were often at least as large as postnatal (Lin et al., 2024; Tusa et al., 2025). Mutual adjustment was not consistently reported, but where timing was distinguished, the pattern is compatible with additive or persistent effects across the perinatal period rather than a purely postnatal explanation.

Narrative studies also highlight heterogeneity in outcomes. One study of developmental domains found that children of mothers with PMADs were significantly more likely to exhibit mild language and motor delays rather than severe developmental delays, with risk intensifying when mothers had both depression and anxiety pre-delivery (Connor et al., 2022). Analysis of cord blood samples from mothers with prenatal depression or posttraumatic stress disorder identified increased expression of ASD**-**associated (Breen et al., 2018) genes, which were linked to reduced cognitive performance in infants at 2 years, suggesting that maternal PMADs may shape early brain development through molecular pathways.

Studies on OCD remain rare, and available evidence is limited. A Taiwanese population study reported a strong association between maternal OCD within 4 years before delivery and offspring ASD (adjusted OR 3.42, 95% CI 1.77–6.63; Chien et al., 2023; Yu et al., 2022), though based on very small numbers (12 cases, 36 controls) and therefore requiring replication.

Several observational studies initially suggested an association between ASD and antenatal antidepressant exposure, but these studies lacked adequate controls (Brown et al., 2017; Harrington et al., 2014; Seebeck et al., 2024; Siracusano et al., 2021; Sørensen et al., 2013; Zhou et al., 2018). Evidence of potential parental rater bias further complicates interpretation: in one Dutch cohort, SSRI exposure (El Marroun et al., 2014) appeared related to autism symptoms only when both parents provided reports, and not when fathers reported alone. Together, these findings indicate that apparent antidepressant–ASD associations may reflect methodological artifacts or underlying maternal illness, rather than a direct pharmacological effect.

Evidence specific to ID was limited in our dataset. Most eligible studies focused on ASD or combined ID with broader developmental outcomes, where ASD was stratified by ID, and associations were observed for ASD without ID but not for ASD with ID, with wide confidence intervals for ID-stratified estimates (Rai et al., 2013).

### Paternal PMAD and the risk of offspring NDDs

**Figure 3.**
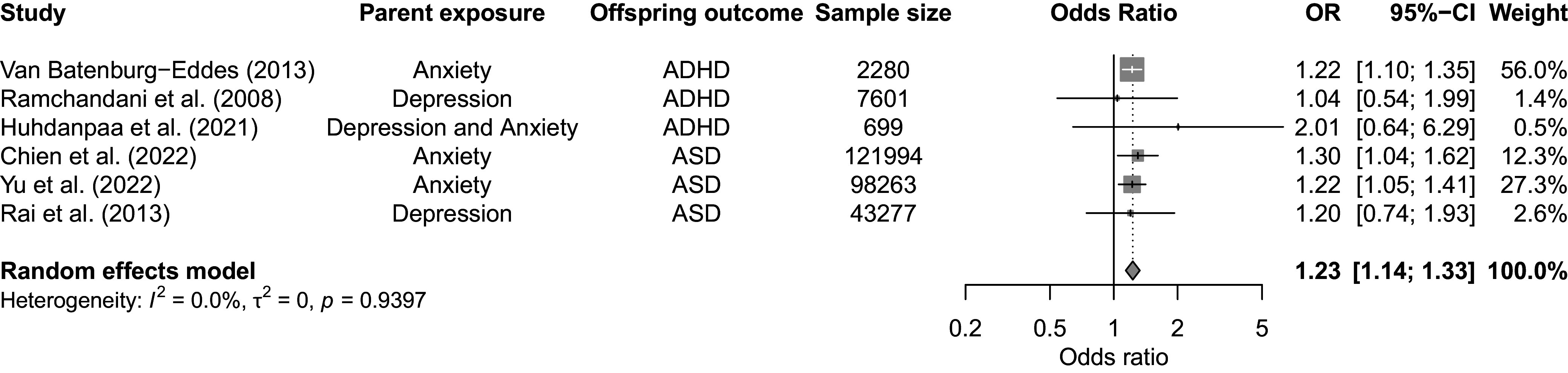
Forest plot for the association between paternal prenatal mood and anxiety disorders and the risk of neurodevelopmental disorders in offspring. *Note*: Square sizes reflect the weights attributed to each study. The diamond denotes the summary effect size for the random-effects models. CI, confidence interval; ASD, autism spectrum disorder; ADHD, attention-deficit/hyperactivity disorder. Chien et al. (2023) included the following disorders: major depressive disorder; persistent depressive disorder; depressive disorder, unspecified; generalized anxiety disorder; panic disorder; agoraphobia; social anxiety disorder; and specific phobia disorder.

Our meta-analysis of six studies identified a significant association between paternal PMADs and offspring NDDs (OR = 1.23, 95% CI 1.14–1.33, *p* < 0.001; Figure 3). Given the few available studies, it was not possible to conduct separate meta-analyses on the effects of paternal PMADs on ADHD and ASD. Studies showed low heterogeneity (Cochran’s *Q* = 1.36, *p* = 0.94, *I* ^2^ = 0%) with no outliers.

The funnel plot (Supplementary Figure S4) showed some asymmetry, although the small number of studies limits formal testing for publication bias. The trim-and-fill method (Supplementary Figure S5) indicated at least two missing studies, suggesting unreported results that could affect the overall effect estimate.

#### Effects of paternal PMAD on ADHD in offspring

Chen et al. (2020) showed that paternal prenatal depression increases offspring ADHD risk, particularly in cases of chronic depression or when both parents are affected. In contrast, Ramchandani et al. (2008) found no significant association between paternal depression during pregnancy and offspring ADHD diagnoses. Other studies also reported limited evidence for an association between prenatal paternal depression, anxiety, and risk of offspring ADHD (Huhdanpää et al., 2021; O’Donnell et al., 2017; Van Batenburg-Eddes et al., 2013).

Adding to this complexity, a single study found that, after controlling for shared genetics, prenatal maternal depression was associated with a slightly elevated offspring ADHD risk, whereas prenatal paternal depression showed a minor association with lowered offspring ADHD risk (Eilertsen et al., 2021). Nonetheless, researchers cautioned against drawing definitive conclusions from this association, noting the lack of theoretical models that explain the effect of paternal prenatal depression. In light of their findings, they also questioned the validity of selecting parents with depression as negative controls for one another in future studies.

#### Effects of paternal PMADs on offspring ASD

Chen et al. (2020) found that offspring ASD risk was elevated when either parent was affected, with a slightly greater risk for paternal than maternal prenatal depression. Additionally, a separate study based on Taiwanese registers found no significant associations between paternal prenatal OCD and offspring ASD, likely due to small sample sizes (Chien et al., 2023; Yu et al., 2022).

### Sex or gender differences

Few studies addressed interactions between offspring sex or gender, NDDs, and maternal and/or paternal PMADs. Of these few, none qualified for meta-analysis. The influence of prenatal maternal and paternal PMADs on NDDs appears to differ by sex, although the existing research is limited and yielded mixed results. The studies did not distinguish between sex and gender.

Loomans et al. (2011) observed that maternal prenatal anxiety significantly correlated with increased hyperactivity and inattention problems in boys but not in girls, suggesting potential sex-specific vulnerabilities to prenatal anxiety. Similarly, Huhdanpää et al., 2021 found that maternal prenatal depressive symptoms were linked to increased inattentiveness and hyperactivity among boys at age 5. These findings imply that boys might be particularly susceptible to the neurodevelopmental influence of prenatal maternal affective symptoms. However, Bendiksen et al. (2020) reported that the higher prevalence of ADHD symptoms, particularly the hyperactive/impulsive subtype in boys, does not reflect a differential contribution of maternal distress between the sexes. This suggests that although boys generally display more ADHD symptoms, the effects of maternal distress during pregnancy affect both sexes similarly. Supporting this lack of sex-specific associations, Chen et al. (2024) found no sex differences in the association between prenatal depression and child mental health outcomes. They suggested that earlier findings indicating that prenatal maternal stress predicts sex-specific child outcomes may vary depending on the particular behavior being examined.

Studies found no sex/gender differences following exposure to maternal prenatal depression and autism-related traits or behavioral problems in offspring (Chen et al., 2024). No studies specifically addressed paternal PMADs.

### Risk of bias

According to assessments conducted with the Newcastle–Ottawa scales, the majority of studies on parental PMADs were at moderate risk of bias (36 studies, 59%), 5 studies (8%) were at low risk, and the remaining 20 studies (33%) were at high risk. Among studies assessed with JBI checklists, two out of three studies reported key information and used correct statistical analyses (Supplementary Table S2).

## Discussion

This systematic review and meta-analysis examined the relationship between PMADs in parents and the risk of NDDs in their offspring. We found a stronger association for maternal PMADs (OR = 1.78, 95% CI 1.53–2.07) than paternal PMADs (OR = 1.24, 95% CI 1.15–1.34). Although not explored in this study, prior research suggests that this larger association with maternal PMADs might be attributed to more direct biological and environmental effects during pregnancy (Feldman et al., 2013; Le Bas et al., 2022), although multiple mechanisms likely contribute. Beyond prenatal exposures, the observed associations might also reflect the influence of postnatal maternal mental health, as many women experience mood and anxiety disorders beyond childbirth (Putnick et al., 2020; Tucker & Hobson, 2022). Indeed, several studies in our review found that adjusting for postnatal symptoms attenuated but did not eliminate prenatal associations, suggesting both prenatal and postnatal periods contribute to offspring risk (Lin et al., 2024; Tusa et al., 2025; Van Batenburg-Eddes et al., 2013; Wolford et al., 2017). These observational associations should be interpreted as statistical relationships rather than causal effects.

In the maternal meta-analysis of 20 articles, we observed low heterogeneity after excluding three studies (Nidey et al., 2021; Seebeck et al., 2024). Potential sources of heterogeneity include variation in sample sizes, diverse PMAD and NDD measurement tools, diverse timeframes between conception and delivery, and few studies accounting for prenatal treatment initiation (Maselko et al., 2015).

Our meta-analysis revealed a strong association between maternal depression and anxiety and an increased risk of ADHD in offspring. The narrative synthesis underscored that specific timing or trajectory of maternal disorder might have varying effects on offspring ADHD. Notably, maternal anxiety during early pregnancy (12–22 weeks) significantly predicted ADHD symptoms (Van den Bergh & Marcoen, 2004). This timing-specific pattern is intriguing, as a purely genetic link might be expected to yield consistent effects across pregnancy, although alternative explanations including timing of symptom ascertainment and gene–environment interactions during specific developmental windows cannot be ruled out. Additionally, a single study highlighted a positive association between maternal prenatal depression symptoms and offspring ADHD risk after adjusting for genetic factors (Eilertsen et al., 2021). Together, these findings suggest that prenatal environment may contribute to the association between PMADs and offspring ADHD. The meta-analysis also revealed a strong association between maternal depression and anxiety and an increased risk of offspring ASD (Chen et al., 2020; Gao et al., 2015; Say et al., 2016).

Studies regarding prenatal OCD were limited and found an association between prenatal maternal OCD and offspring ASD (Yu et al., 2022). Studies of offspring ID were also scarce and either analyzed combined ID and NDDs or ID-related symptoms. These studies reported that offspring of mothers with PMADs were at greater risk of mild language and motor delay or gross motor deficits (Lupattelli et al., 2019; Wiggins et al., 2019).

Finally, current research addressing the association of parental PMADs on sex/gender differences in offspring NDDs is inconsistent. Some studies suggest a tentative association with ADHD symptoms in boys, but not girls, and no significant gender/sex associations with ASD or autism-related traits (Avalos et al., 2023; Bendiksen et al., 2020; Huhdanpää et al., 2021; Loomans et al., 2011). These differences may stem from gender- or sex-specific differences in how parental mental health influences child outcomes, methodological differences between studies, or cultural patterns that influence diagnosis. For instance, the ADHD symptom of hyperactivity may be more disruptive than inattention in certain socioeconomic and healthcare environments, leading to higher diagnostic rates in boys. Alternatively, diagnostic rates may be affected by gender role expectations or stereotypes of neurodivergent presentations in individuals with different gender identities. Another explanation may be that more recent studies used larger samples, including testing multiple cohorts (Avalos et al., 2023; Shuffrey et al., 2023). Patterns of results may differ between diverse populations.

**Figure 4.**
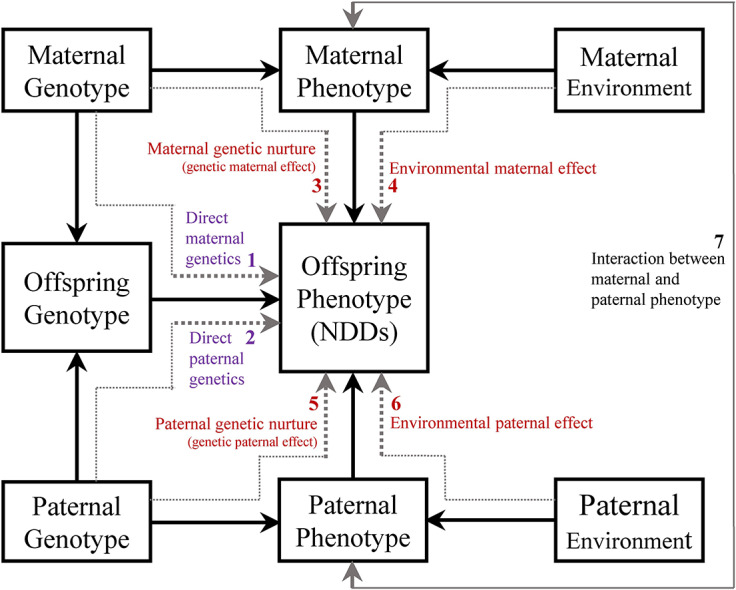
Maternal and paternal effect on offspring phenotype (a neurodevelopmental disorder). It includes three main pathways: the maternal/paternal genetic nurture effect, showing transmission from maternal/paternal genotype to their respective phenotypes and then to the offspring’s phenotype (dashed arrows 3 and 5); the environmental maternal/paternal effect, indicating the influence of the maternal or paternal environment on their phenotype and consequently on the offspring’s phenotype (dashed arrows 4 and 6); and the direct maternal/paternal genetic effect, which traces direct transmission from maternal/paternal genotype to the offspring’s genotype and then to their phenotype (dashed arrows 1 and 2). This figure omits child environment effects or gene and environment interactions.

Multiple pathways are likely involved in the association between maternal PMADs and the risk of NDDs in offspring. The first pathway (Figure 4, arrow 1) involves direct transmission of genes from the mother to the offspring. Recent genomic studies indicate this genetic transmission involves pleiotropic effects, where the same genetic variants increase risk for both parental psychiatric conditions and offspring neurodevelopmental conditions (Chen et al., 2024; Eilertsen et al., 2021; Shakeshaft et al., 2023). In some cases, reverse causation may occur where parental NDD traits manifest first and contribute to secondary mood and anxiety symptoms, with offspring inheriting the underlying NDD genetic risk rather than being affected by parental mood symptoms per se. The second and third pathways (arrows 3 and 4), are known as *maternal effect* (Wolf & Wade, 2009). Maternal effect refers to the impact of the mother’s phenotype on the offspring’s phenotype, above and beyond the transmission of maternal genes: via environmental pathways instead of directly transmitted genetic risk. Maternal effect can arise from maternal genotype (maternal genetic effect, also called maternal genetic nurture; arrow 3), and/or maternal environment (environmental maternal effect; arrow 4). Paternal PMADs are also associated with the risk of offspring NDD via three analogous pathways (arrows 2, 5, and 6).

Maternal/paternal effects have been defined as causal impacts of the maternal/paternal genotype or phenotype on the offspring phenotype (Wolf & Wade, 2009). However, statistical models used for estimating parental effects may not always provide causal estimates due to several factors: (1) *Maternal/paternal effects could depend on offspring genotypes*, such as an additive-by-additive interaction between the maternal/paternal and the offspring genotype (Wolf & Wade, 2009). For instance, an interaction between maternal anxiety and the offspring gene may predict the severity of ADHD symptoms, suggesting an additive-by-additive interaction between the maternal and offspring genotypes (O’Donnell et al., 2017); (2) *Lack of blinding in observational family-based studies* may lead to diagnostic bias, as relatives of probands with psychiatric disorders may be more likely to be diagnosed, due to family history being part of the diagnostic process. This could result in overestimated genetic influence in family-based studies; (3) *Assortative mating* (nonrandom mating) can influence population prevalence estimates and estimates of direct genetic effect; (4) *Selection bias* due to differential access to healthcare, where individuals with milder disorder forms or who do not seek or cannot access clinical services may be excluded from research; (5) *Gene–environment interactions can complicate causal inference* (they were omitted in Figure 4). Several studies reviewed here suggested such mechanisms, for example, for offspring ADHD, depression or anxiety interacting with smoking during pregnancy, prenatal infection with prenatal anxiety, and short breastfeeding in mothers with prenatal depressive mood or anhedonia (Bendiksen et al., 2020; Koutra et al., 2017; O’Donnell et al., 2017; Say et al., 2016); (6) *Measurement errors* can introduce uncertainties and biases into the analysis; (7) *Failure to achieve exchangeability between exposed and unexposed groups* may lead to confounding, undermining the validity of causal conclusions; and (8) *Retrograde effects*, where the offspring phenotype may influence parental phenotype. Examples include fetomaternal immune incompatibility, such as Rh factor incompatibility, which could lead to maternal depressive symptoms and later cognitive effects in the offspring.

### Strengths and limitations

This study has several important strengths. First, our preregistered protocol ensures transparency and reduces selective reporting bias. Second, we conducted the most comprehensive synthesis to date, analyzing 21 studies in meta-analysis and 53 additional studies in narrative review, encompassing multiple NDDs (ADHD, ASD, and ID) rather than focusing on single outcomes. Third, we included both maternal and paternal PMADs, addressing a critical gap as most prior reviews focused solely on maternal effects. Fourth, our analytical approach was rigorous, including sensitivity analyses to identify outliers, assessment of heterogeneity, and examination of publication bias. Finally, our narrative synthesis captured nuanced findings about timing effects, sex/gender differences, and trajectory patterns that would be missed by meta-analysis alone.

However, this study also has important limitations. First, several methodological constraints affect our analyses. The meta-analysis of paternal PMADs included only 7 studies compared with 20 for maternal PMADs, resulting in less statistical power for detecting paternal effects. Inconsistent reporting of postnatal adjustment across primary studies precluded stratified meta-analysis by postnatal control, limiting our ability to isolate prenatal from postnatal effects. Effect sizes varied substantially by informant (parent vs. teacher ratings) and outcome definition (symptoms vs. clinical diagnosis), contributing to heterogeneity and limiting cross-study comparability. Furthermore, most studies did not account for prenatal treatment, making it impossible to disentangle PMAD effects from medication effects.

Second, generalizability is constrained by population characteristics. Studies predominantly represented Western, high-income countries, with limited representation from Africa, South America, and low/middle-income settings. None provided explicit definitions of mothers and fathers, precluding generalizability to transgender or nonbinary parents, coparents, or nonbiological partners (Nilsen et al., 2009). Most studies lacked detailed breakdowns of ethnicity, race, or ancestry. Several studies drew from the same cohorts (particularly Scandinavian registers), and although we selected the largest samples for meta-analysis, this may overrepresent specific populations.

Third, we could not address several important clinical questions due to data limitations. Few studies examined specific PMAD timing, severity, or chronicity. Studies rarely distinguished between PMAD subtypes, potentially masking disorder-specific associations. Finally, limited data on OCD and ID prevented robust conclusions about these outcomes.

### Conclusion

Our comprehensive meta-analysis demonstrates associations between parental PMADs and offspring NDDs, with stronger associations for maternal PMADs (pooled OR = 1.78) than for paternal PMADs (pooled OR = 1.24). These associations likely reflect both inherited genetic factors and environmental pathways, including the behavioral and developmental impacts of parental mental health on family functioning.

Despite mechanistic uncertainty, the association itself has important implications for clinical practice and family support. Regardless of underlying pathways, families affected by parental PMADs represent an enriched risk group who may benefit from enhanced access to developmental support services and early intervention resources. This family-centered approach prioritizes both parental mental health and child development, offering proactive support rather than waiting for difficulties to emerge. Importantly, although these children have elevated risk for NDDs, most will not develop these conditions, and support should be framed as promoting optimal development rather than preventing inevitable outcomes.

## Supporting information

10.1017/S0033291725103139.sm001 Kępińska et al. supplementary material Kępińska et al. supplementary material

## Supplementary material

The supplementary material for this article can be found at http://doi.org/10.1017/S0033291725103139.
